# An App Detecting Dengue Fever in Children: Using Sequencing Symptom Patterns for a Web-Based Assessment

**DOI:** 10.2196/11461

**Published:** 2019-05-31

**Authors:** Tsair-Wei Chien, Julie Chi Chow, Willy Chou

**Affiliations:** 1 Data Analyses & Statistics Medical Research Chi-Mei Medical Center Tainan Taiwan; 2 Pediatrics Chi-Mei Medical Center Tainan Taiwan; 3 Physical Medicine and Rehabilitation CHia-Li Campus Chi-Mei Medical Center Tainan Taiwan; 4 Department of Recreation and Health-Care Management Chia Nan University of Pharmacy Tainan Taiwan

**Keywords:** dengue fever, HT person mapping statistic, logistic regression, score summation, receiver operating characteristic curve

## Abstract

**Background:**

Dengue fever (DF) is one of the most common arthropod-borne viral diseases worldwide, particularly in South East Asia, Africa, the Western Pacific, and the Americas. However, DF symptoms are usually assessed using a dichotomous (ie, absent vs present) evaluation. There has been no published study that has reported using the specific sequence of symptoms to detect DF. An app is required to help patients or their family members or clinicians to identify DF at an earlier stage.

**Objective:**

The aim of this study was to develop an app examining symptoms to effectively predict DF.

**Methods:**

We extracted statistically significant features from 17 DF-related clinical symptoms in 177 pediatric patients (69 diagnosed with DF) using (1) the unweighted summation score and (2) the nonparametric *HT* person fit statistic, which can jointly combine (3) the weighted score (yielded by logistic regression) to predict DF risk.

**Results:**

A total of 6 symptoms (family history, fever ≥39°C, skin rash, petechiae, abdominal pain, and weakness) significantly predicted DF. When a cutoff point of >–0.68 (*P*=.34) suggested combining the weighted score and the *HT* coefficient, the sensitivity was 0.87, and the specificity was 0.84. The area under the receiver operating characteristic curve was 0.91, which was a better predictor: specificity was 10.2% higher than it was for the traditional logistic regression.

**Conclusions:**

A total of 6 simple symptoms analyzed using logistic regression were useful and valid for early detection of DF risk in children. A better predictive specificity increased after combining the nonparametric *HT* coefficient with the weighted regression score. A self-assessment using patient mobile phones is available to discriminate DF, and it may eliminate the need for a costly and time-consuming dengue laboratory test.

## Introduction

### Symptoms of Dengue Fever

Dengue fever (DF) is one of the most common arthropod-borne viral diseases worldwide [1], especially in South East Asia, Africa, the Western Pacific, and the Americas [2,3].

However, there is no accurate and speedy diagnostic screening test for DF at an early stage, as its signs and symptoms—for example, fever, headache, and myalgia—are similar to those of other illnesses [4-6]. Some studies [4,5] that used a univariate analysis report that the presumptive diagnosis of DF is imprecise. Multivariate logistic regressions also do not significantly distinguish patients with dengue from those with other febrile illnesses [7]. The multivariate discrimination analyses reported sensitivity and a specificity 0.76 and an area under the receiver operating characteristic (ROC) curve (AUC) of 0.93, but costly laboratory tests (Dengue Duo Immunoglobulin M and Rapid Strips, Panbio, Queensland, Australia) [8-11] were needed before DF was serologically confirmed.

### Assessment of Dengue Fever

DF symptoms are usually assessed using a dichotomous (ie, absent vs present) evaluation. The dependent variable (DF^+^ vs DF^−^) predicted using independent evaluations with a weighted summation score is more accurate than that predicted using simple evaluations with an unweighted summation score. So far, there has been no published study that has reported using the specific sequence of symptoms reported or observed in specific patients suspected of having DF. All published studies to date still report results using only a standard group of symptoms with an unweighted summation score, and they merely apply their results to a general group of patients who might have DF.

### The HT Fit Statistic Applied to Detect Dengue Fever

The nonparametric HT fit statistic has been used in education and psychometrics to identify aberrant test respondents [12,13]. It is a transposed formulation of a scalability coefficient for items (eg, symptoms in this study), and it is the best among 36-person fit statistics for detecting abnormal behaviors [14].

### Objectives

In this study, we used the *HT* coefficient combined with weighted and unweighted variables to examine whether these combinations provide a valid and reliable approach for the early detection of DF in children.

## Methods

### Sample and Clinical Symptoms

The sample of 177 pediatric patients (≤16 years old; DF^+^: 69; DF^−^: 108) was the same as in our previous paper [8] (see data in Multimedia Appendix 1). Guided by the literature [5-7], we collected 19 DF-related clinical symptoms from the patients’ medical records to develop the initial set of items—designated as 0=“absent” or 1=“present”—to screen for DF infection: (1) personal history of DF, (2) family history of DF, (3) mosquito bites within the previous 2 weeks, (4) fever ≥39°C, (5) biphasic fever, (6) rash, (7) petechiae, (8) retroorbital pain, (9) bone pain (arthralgia), (10) headache, (11) myalgia, (12) abdominal pain, (13) anorexia, (14) occult hematuria, (15) stool occult blood, (16) cough, (17) sore throat, (18) soft (watery) stool, and (19) flushed skin. Data from these patients’ charts were obtained and approved by the Research Ethics Review Board of the Chi-Mei Medical Center.

### The HT Fit Statistic

*HT* is defined for the persons of a dichotomous dataset with *L* items (in columns) and *N* persons (in rows) [12-14], where *X*_ni_ is the scored (0,1) response of person *n* to item *i*, and *P*_n_=*S*_n_*/L*. Here, *S*_m_ is the raw score for person *m*, and *S*_n_ is the raw score for person *n*.

**Figure 1:**
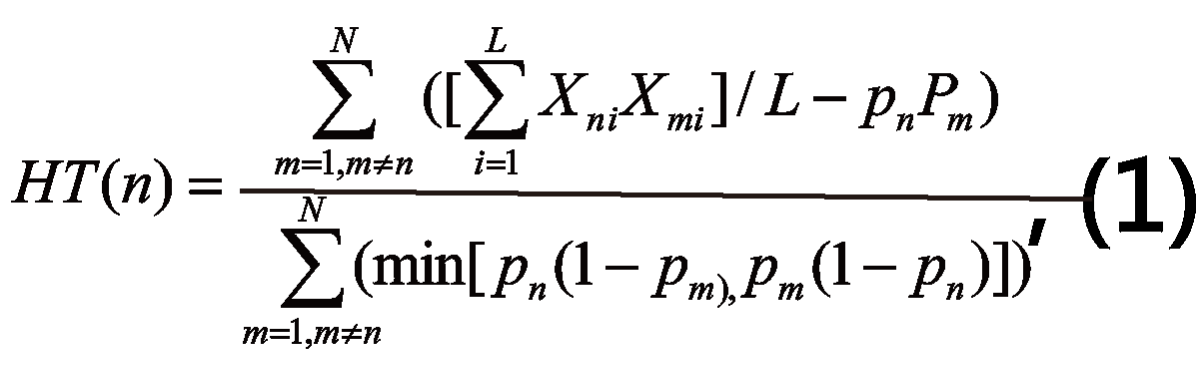
The equation of the HT fit statistic.

*HT* is the sum of the covariances between person *n* and the other persons divided by the maximum possible sum of those covariances so that the range of *HT* is from −1 to +1, see formula (1) in Figure 1. When the responses by person *n* are positively correlated with those of all the other persons, then *HT* (*n*) will be positive. In contrast, when the responses by person *n* are negatively correlated with those of all the other persons, then *HT* (*n*) will be negative. When person *n* ’s responses are random, *HT* (*n*) will be close to zero [11]. We hypothesized that DF^+^ patients have different *HT* coefficients than DF^−^ patients. All DF^+^ group members were sequenced to the DF^−^ group members to obtain an *HT* coefficient using formula (1) in Figure 1.

### Selecting Symptoms and Determining Predictor Variables

**Figure 2:**
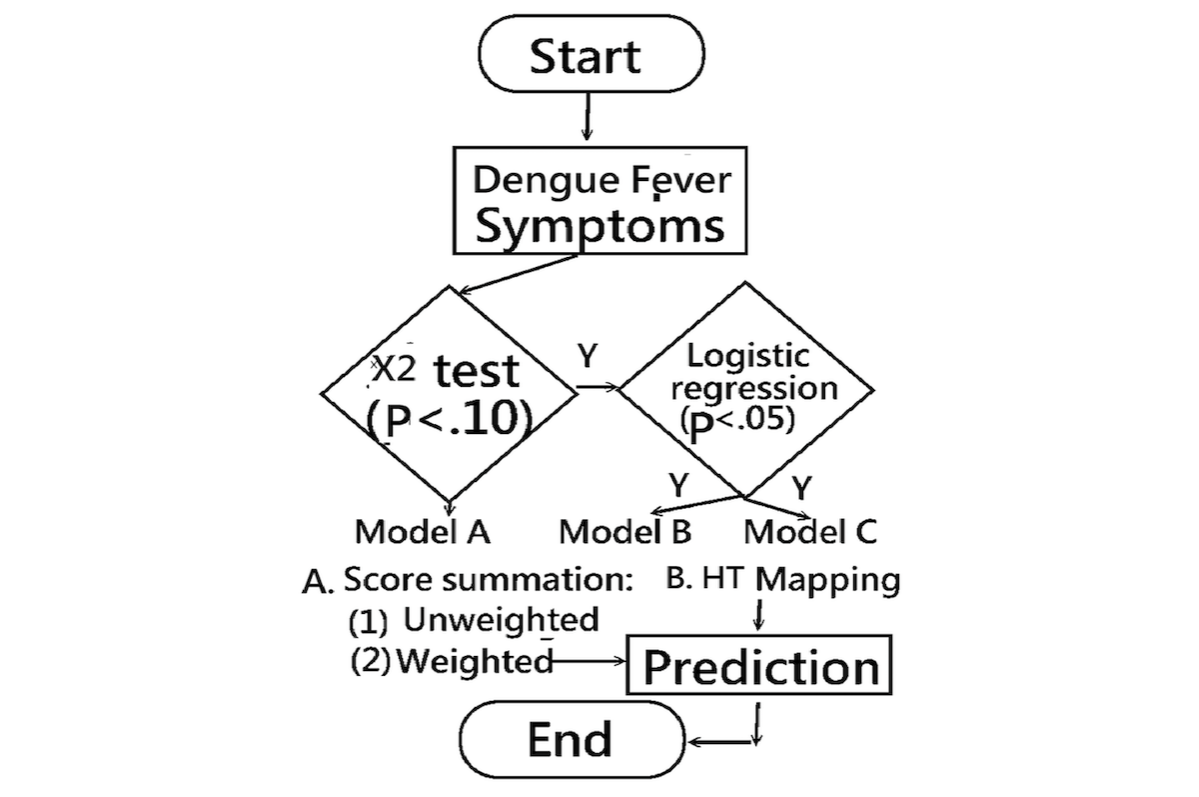
Overall study concept and the flow chart.

All symptoms were examined by the probability of Type 1 error using the following 3 steps in Figure 2 to determine predictor variables. First, each symptom was separately examined by the univariate approach using a Chi-square test and logistic regression, respectively, for identifying a significant association with DF. Second, 2 models (ie, the univariate and the multivariate approaches) were investigated for determining valid predictor variables associated with DF when the probability of Type 1 error was less than .05. Third, the predictor variables were used in a weighted combination for discriminating patients suspected with dengue virus infection.

### Detecting Dengue Fever: A Comparison of Three Models

The efficacy of 3 models (A, B, and C) for detecting dengue fever was examined: (1) A comparison was made using univariate logistic regression in Model A to examine effects through the AUC, yielded by unweighted (ie, summed item) scores, weighted (ie, logistic regression) scores, and *HT* coefficients, respectively. (2) Multivariate logistic regression with the 3 aforementioned factors combined was used in Model B. (3) After selecting the significant variables in Model B, the combined predictive variables were analyzed using multivariate logistic regression in Model C to obtain effective weighted coefficients. (4) Finally, we wanted to use a single continuous variable yielded by the combined predictive variables in Model C to compare the AUC with the counterparts in Model A and C.

Moreover, we provide the F-measure for evaluating the predictive effect [15], which is calculated by following equations: precision=True Positives/(True Positives+False Positives); recall=True Positives/(True Positives+False Negatives); F-measure=(2×precision×recall)/(precision+recall).

### Statistical Tools and Data Analyses

SPSS 15.0 for Windows (SPSS Inc) and MedCalc 9.5.0.0 for Windows (MedCalc Software) were used to calculate (1) the probability of false positives (Type 1 error) using a Chi-square test and logistic regression, (2) Youden J index (the higher, the better), AUC, sensitivity, specificity, and the cutoff point at maximal summations of specificity and sensitivity, (3) correlation coefficients among variables of unweighted, weighted, and *HT* scores.

## Results

### Demographic Characteristics of the Study Sample and the Likelihood of Dengue Fever

A total of 69 pediatric patients clinically diagnosed with DF and 108 pediatric patients with no evidence of DF infection were included in this study (Table 1). A Chi-square test and logistic regression analyses showed that only 6 symptoms (family history, fever ≥39°C, skin rash, petechiae, abdominal pain, and weakness) were significant for assessing the likelihood of DF (Table 2).

**Table 1:** Demographic characteristics of the study sample.

Demographical variables		Dengue fever (–)^a^, n (%)	Dengue fever (+)^b^, n (%)	Total, n (%)	*P* value^c^
**Gender**					
	Female	47 (43.5)	29 (42)	76 (42.9)	.84
	Male	61 (56.5)	40 (58)	101 (57.1)	—^d^
**Age (years)**					
	0-4	48 (44.4)	11 (16.2)	59 (33.5)	.005
	5-9	24 (22.2)	20 (29.4)	44 (25)	—
	9-16	36 (33.3)	37 (54.4)	73 (41.5)	—

^a^Dengue fever (–): patients with a negative dengue fever strip test.

^b^Dengue fever (+): patients with a positive dengue fever strip test.

^c^*P* values were determined by the Chi-square test.

^d^Not applicable.

**Table 2:** Logistic analysis of symptoms for the patients suspected with dengue virus infection using the univariate approach.

Symptom variables and presence		Dengue fever (–)^a^, n (%)	Dengue fever (+)^b^, n (%)	Total, n (%)	Chi-square (df)	*P* value^c^	Logistic regression	
							Beta	*P* value
**Family history**								
	No	79 (73.1)	40 (58.0)	119 (67.2)	3.7(2)	.053	1.35	.002
	Yes	29 (26.9)	29 (42.0)	58 (32.8)	—^d^	—	—	—
**High fever of 39°C**								
	No	87 (80.6)	37 (53.6)	124 (70.1)	13.3(2)	<.001	1.48	.048
	Yes	21 (19.4)	32 (46.4)	53 (29.9)	—	—	—	—
**Skin rash**								
	No	82 (75.9)	20 (29.0)	102 (57.6)	36.1(2)	<.001	2.63	.000
	Yes	26 (24.1)	49 (71.0)	75 (42.4)	—	—	—	—
**Petechiae**								
	No	106 (98.1)	60 (87.0)	166 (93.8)	7.3(2)	.007	2.34	.026
	Yes	2 (1.9)	9 (13.0)	11 (6.2)	—	—	—	—
**Abdominal pain**								
	No	104 (96.3)	53 (76.8)	157 (88.7)	14.1(2)	<.001	2.89	.000
	Yes	4 (3.7)	16 (23.2)	20 (11.3)	—	—	—	—
**Weak sense**								
	No	90 (83.3)	48 (69.6)	138 (78.0)	3.9(2)	.049	0.98	.048
	Yes	18 (16.7)	21 (30.4)	39 (22.0)	—	—	—	—
**Constant**	
	—	—	—	—	—	—	–3.28	—

^a^Dengue fever (–): patients with a negative dengue fever strip test.

^b^Dengue fever (+): patients with a positive dengue fever strip test.

^c^*P* values were determined by the Chi-square test and the Wald test of logistic regression.

^d^Not applicable.

### Comparisons of the Area Under Receiver Operating Characteristic Curve for the Three Study Models

Comparisons of the AUCs for the 3 study models (A, B, and C) showed that the weighted variable (derived by the Logistic regression) and the *HT* coefficient could be jointly used for predicting DF risk using equation (2):

(
*Logit*=−3.32+0.93 x
*weighted* _
*score* + 1.92 × HT ¬_
*coefficient*) (2)

The risk probability can be computed using the transformed formula 3:

P=exp (log
*it*)/ (1+exp(log
*it*)) (3)

where *logit* denotes a unit of log odds.

**Figure 3:**
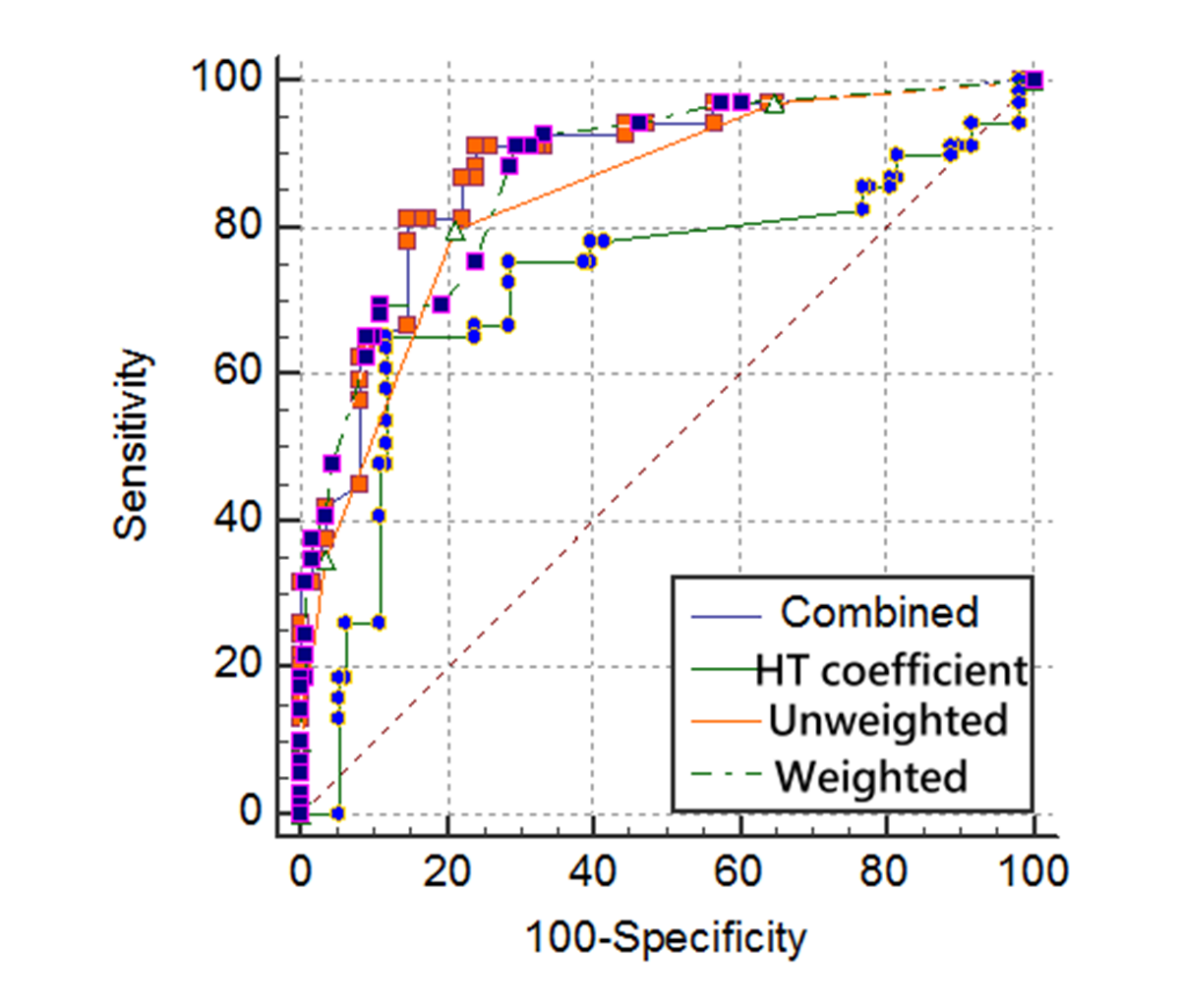
Four models plotted by receiver operating characteristic curves. The Combined denotes Model C in this study (sensitivity=0.87, specificity=0.84, area under the receiver operating characteristic curve=0.91, F-measure=0.82).

A cutoff point of >–0.68 (*P*=.34) was determined using the combined predictive variables in Model C: sensitivity=0.91, specificity=0.76, AUC=0.88, and the highest F-measure=0.82 (see Figure 3 and Table 3). Predictive power was better: specificity was 10.2% (ie, 84.30–74.10, shown in Table 3) higher than when using traditional logistic regression, *that is, the independence variable=sum (weighted score for each symptom* x *the respective symptom response, 1 or 0, predicting the dependence variable, 1 or 0 for DF*). Even if AUC using the *HT* coefficient was slightly lower (0.72) than when using the unweighted (0.84) and the weighted (0.87) variables (*Table* *3*), and the *HT* coefficients related to the weighted and unweighted scores were 0.26 and 0.22, respectively, the weighted score had a higher correlation coefficient than the unweighted score to the *HT* coefficients, and the combined strategy of Model C or the single continuous variable yielded by the combined predictor variables (Table 3) are verified and available for use in practice. *More importantly, the sensitivity is more critical than the specificity in clinical settings, as we would not miss any 1 case with fatal diseases.*

**Table 3:** Comparisons of area under receiver operating characteristic curve for the study models.

Approach and steps				Logistic regression		Receiver operating characteristic curve analysis					F-measure
				B^a^	*P* value	Area under receiver operating characteristic curve	Youden J^b^	Cut point	Sensitivity	Specificity	
**Comparison of models**											
	**Model A:** *Univariate approach with a single variable compared with the dengue fever using Logistic regression and receiver operating characteristic* *analysis*										
		Unweight^c^		1.60^d^	<.001	0.84	0.58	>1.00	79.7	78.7	—^e^
		Weight^f^		0.97^d^	<.001	0.89	0.61	>–1.20	91.3	74.1	—
		HT coefficient^g^		3.75^d^	<.001	0.72	0.53	>0.15	65.2	88	—
	**Model B:** *Multivariate approach with combined these three variables in regressing the dengue fever using Logistic regression*										
		Unweight		0.31	.595	—	—	—	—	—	—
		Weight		0.77^d^	.014	—	—	—	—	—	—
		HT coefficient		3.08^d^	.001	—	—	—	—	—	—
		Constant		–1.03	.35	—	—	—	—	—	—
	**Model C** *: Combined these 2 significant predictor variables using Logistic regression*										
		Weight		0.919^d^	<.001	—	—	—	—	—	—
		HT coefficient		2.962^d^	.001	—	—	—	—	—	—
		Constant		–0.463	.751	—	—	—	—	—	—
	**A single continuous variable** *yielded by the combined predictor variables in Model C*										
		Combined^h^		1	<.001	0.91	0.71	>–0.68	87	84.3	—
**The predictive effect: precision recall**											
	Unweight			—	.72	0.85	—	—	—	—	0.78
	Weight			—	.93	0.65	—	—	—	—	0.77
	HT coefficient			—	.78	0.82	—	—	—	—	0.8
	The combined model			—	.87	0.78	—	—	—	—	0.82

^a^B: coefficient of logistic regression.

^b^Youden J index.

^c^Item-score summation method.

^d^*P*<.05.

^e^Not applicable.

^f^Multiplying item score with the weighted regression coefficient.

^g^See Figure 1 for the HT equation

^h^Using the 2 combined variables to predict patient’s dengue fever.

**Figure 4:**
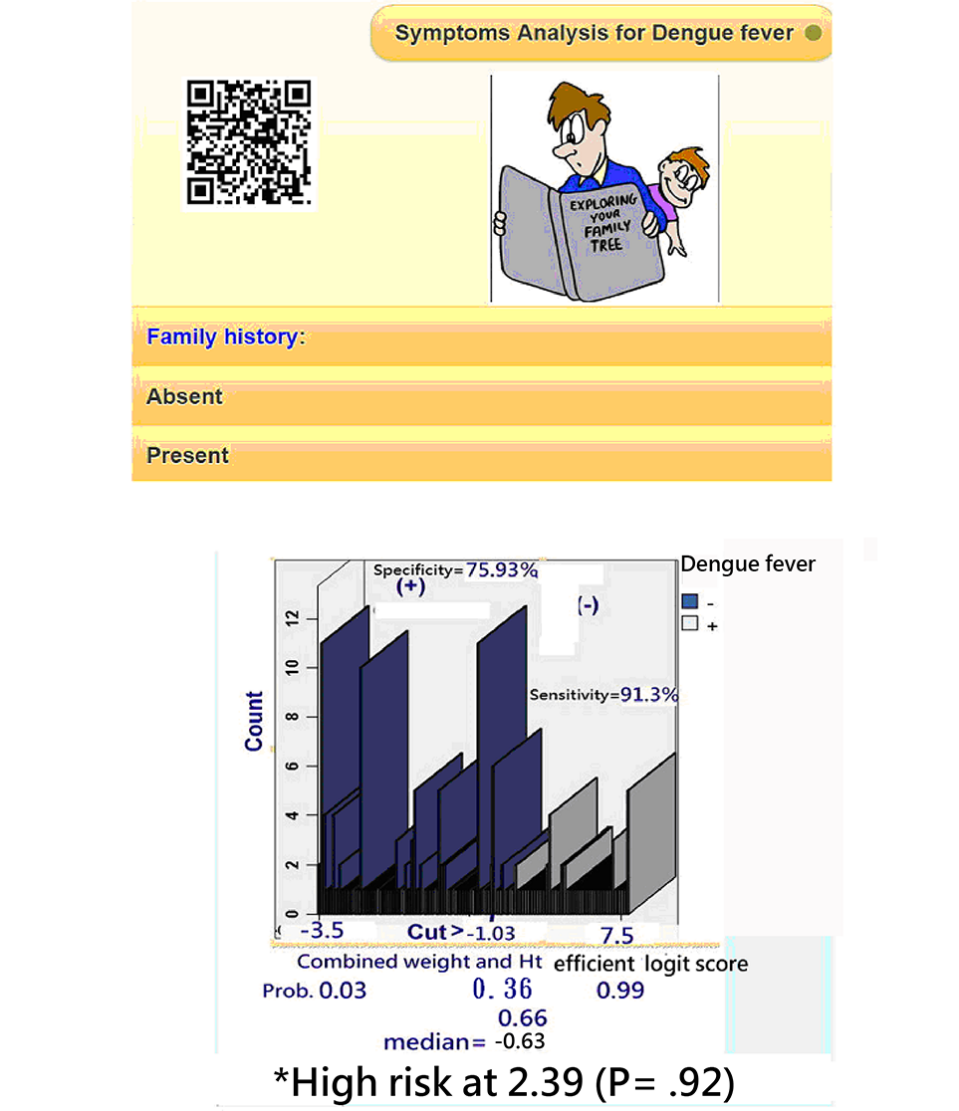
Snapshots on a mobile phone responding questions (top) and the result (bottom) for assessing the patient dengue fever.

A snapshot on a mobile phone responding to questions (Figure 4, top) was generated, and the results for assessing whether the patient has DF (Figure 4, bottom) were determined, which indicated that patients suspected of having DF could directly scan the Quick Response Code to obtain their DF *logit* scores (or the risk probability) and examine whether these 6 symptoms are useful for predicting a high DF risk (>−1.03 *logits* or *P* ≥.26=exp(−1.03 logits)/(1+exp(-1.03 logits)). Interested readers are recommended to see the demonstration in Multimedia Appendix 2 using a MP4 video to display.

## Discussion

### Principal Findings

We found that using the HT coefficient yielded predictions that were 10.2% more specific (ie, 84.30–74.10, shown in Table 3) than those of traditional logistic regression. The *HT* index is promising when the patient sequence symptom pattern is compared with the DF^+^ group to detect dengue fever in children. It can be combined with the weighted summation score to jointly predict the DF risk and then report that risk on mobile phones.

The *HT* coefficient has been used in education and psychometrics to identify aberrant test respondents [12-14]. Although some have used item response theory fit statistics (eg, outfit mean square error >2.0) to select abnormal responses that indicate cheating, careless responding, lucky guessing, creative responding, or random responding [16], our literature review revealed no published papers that reported using the *HT* coefficient in medical settings, especially for detecting individual aberrant response patterns different from the study reference sample, or, like this study, identifying the DF risk by comparing their sequence symptom pattern with that of the DF^+^ group.

### What This Knowledge Adds to What We Already Knew

A diagnosis of DF is usually confirmed by 3 steps: (1) observing DF-related symptoms, (2) testing laboratory data, such as white blood cells and platelets, and (3) serologically verifying DF using dengue Immunoglobulin M and Immunoglobulin G antibodies, polymerase chain reaction analysis, and virus isolation tests [8]. The latter 2 are relatively expensive. It is needed to develop a self-assessment approach (eg, scanning Quick Response Code, responding questions, and obtaining the DF risk on his/her smartphone), (1) helping patients for consultation at an earlier stage and (2) prompting doctors for sampling patient laboratory data when his/her DF risk reaches a cut point of *P*=.26 (=exp(−1.03 logits)/(1+exp(−1.03 logits)).

We found that the weighted score was a better predictor than the unweighted score (see Model A and Model B in Table 3). However, we still see so many scales in a medical setting using unweighted summation scores to determine the presence or absence of disease. Along with the mobile phones popularly used in the technical age, the way of obtaining the DF risk on mobile phones using the combined *HT* coefficient and weighted scores is available and worth recommending to health care providers to use for detecting the risk for DF.

### Limitations and Future Study

This study has some limitations. First, the DF cut point based on the symptoms of this study sample might be biased toward that population. Moreover, we did not remove abnormal data when the *HT* coefficient was less than the critical value of 0.22, which best identifies aberrantly responding examinees [14]. Second, although the sample size was small, using the *HT* coefficient combined with the AUC yielded highly accurate discriminatory screening. However, this finding requires confirmation in prospective studies of other regions with a substantial incidence of DF. Third, the study sample size (=177) is too small to make the inference reliable and supportable. More DF patients collected in a study are required to be considered in the discernable future. Particularly, artificial intelligence (AI) has become increasingly prevalent in recent years.

### Conclusions

Analyzing 6 simple symptoms using logistic regression is useful and valid for the early detection of DF risk in children. Combining the *HT* coefficient with the weighted score yields a prediction that is 10.2% more specific than that yielded by traditional logistic regression. A self-assessment app using patient mobile phones is available to help people suspected of having DF, and it might eliminate the need for costly and time-consuming laboratory tests.

## Appendix

Multimedia Appendix 1 Data for the sample of 177 pediatric patients used in this study.

Multimedia Appendix 2 How to run the check on DF online .

Multimedia Appendix 3 Response to the editors.

## Abbreviations

AI: artificial intelligence
AUC: area under receiver operating characteristic curve
DF: dengue fever
ROC: receiver operating characteristic
