# Supplementary material for: An App Detecting Dengue Fever in Children: Using Sequencing Symptom Patterns for a Web-Based Assessment
Source: JMIR Mhealth Uhealth. 2019 May 31;7(5):e11461. doi: 10.2196/11461 (PMC6658251; doi:10.2196/11461)
Supplement: Multimedia Appendix 3 [file mhealth_v7i5e11461_app3.docx]

Editor/Author Correspondence

Editor

2018-10-08 04:25 PM

Delete

Subject: [JPP ms#11461] Editorial Decision/Comments on "An App Detecting Dengue Fever in Children: Using Sequencing Symptom Patterns for An Online Assessment"

------------------------------------------

Decision C: (revision & re-review)

------------------------------------------

Dear Dr Tsair-Wei Chien,

thank you very much for your recent submission (ms #11461) to the JMIR Pediatrics and Parenting (JPP).

-------------------

EDITORIAL COMMENTS (Decision: C)

-------------------

Common formatting / editorial problems (see also Instructions for Authors!):

\### Further Editorial / Peer-reviewer Comments:

I. Moved to JMIR Pediatrics (where it could be published free of charge), although JMIR Public Health or i-JMR would be other possibilities. Please let us know on revision what you prefer.

Response: We prefer to JMIR Pediatrics

II. Please disclose your relationship to ShihBin Su, as we noticed that you nominate this individual as peer-reviewer for virtually all your submissions and this reviewer is never critical of your work. Are there any COIs you wish to disclose?

For future submissions we would ask to refrain from nominating reviewers from Taiwan, to ensure that the reviews remain unbiased and objective.

Response: We agree your advice that all our nominate should be refrained from Taiwan in the future.

External Peer-Review Report(s)

------------------------------------------

------------------------------------------------------

Reviewer A:

General comments

=============

This is a very interesting article developing an App for detecting Dengue Fever in Children, which is worthy of publication because the analysis and discussion of the psychometric properties of the scale analysis were well done and substantially described as well as illustrated by using online App for the public.

Dengue fever (DF) is one of the most common arthropod-borne viral diseases worldwide, particularly in South East Asia, Africa, the Western Pacific, and the Americas. Authors developed App that helps patients/their family members or clinicians to identify DF at an earlier stage is merit as a successful example for mHealth and uHealth. The sensitivity 0.91 means a substantially high precision in detecting patients positive. In contrast specificity 0.76 indicates a medium level of detecting negative and required further observation and inspection which fits the clinical requirement and expectation in healthcare settings. However, some points must be revised, as suggested as below.

Specific comments

=============

Major comments

---------------------

None.

Minor comments

---------------------

1. The concept of symptoms was applied in this study and fitted to the era of big data. In limitation, authors should add one or more sentences to describe using a large sample, instead of the sample of 177 pediatric patients for this study, in the future to verify the study effective and efficient.

Response: We have added one paragraph(i.e., limitation three) in the revised manuscript for better the article in a logic matter.

2. For instance, the feature of this study is the pattern calculation using H(t) equation to help detect DF. A multimedia file (e.g., MS Excel or MP4) regarding the calculation of H(t)is required to provide for readers and to add the value of this study.

Response: We have added on dataset in Multimedia

3. It is good for readers if an MP4 video regarding the App in process (i..e, scan the QR-code in Figure 3, top) can be provided to readers in this study.

Response: We have recorded one MP4 in Multimedia

4. Under Figure 2, it is better to add a note to interpret the Figure about the Model C as “Note. Combined denotes Model C in this study (sensitivity = 0.91, specificity = 0.76, and AUC = 0.88)” for clarifying the Figure 2 in meaning.

Response: In response to the reviewer’s advice, we have added one footnote beneath Figure 2 to clarify the interpretation.

5. In Results, the sentence of “the AUC was slightly lower (0.72) than when using the unweighted (0.84) and the weighted (0.87) variables (Table 2)” was unclear. Whether it should be “the AUC using the Ht coefficient was slightly lower (0.72) than when using the unweighted (0.84) and the weighted (0.87) variables (Table 2) is required to clarify again.

Response: We have added one sentence in the revised manuscript to emphasize the importance of sensitivity more critical than the specificity in clinical settings.

6. In Results, the sentence of “specificity was 5.56% (i.e., 75.93-70.37 shown in Table 3) higher than when using traditional logistic regression” is unclear about the term of traditional logistic regression. I guess it might be the independence variable = sum (weighted score for each symptom * the respective symptom response, 1 or 0, predicting the dependence variable, 1 or 0 for DF). Authors should add one sentence to interpret it after the original sentence.

Response: We have added one sentence with red color in the paragraph in the revised manuscript.

7. One line in Table 3 to describe the feather about the AUC,Youden Jb, Cut point ,Sensitivity and Specificity in comparison with the above alternative is better in pair-comparison for interpreting the results in context.

Response: We have added one statement for each scenario in Table 3

8. A dataset including the original data and the calculation of H(t) are expected in the revised version of manuscript. Good luck and thanks for providing this interesting work.

Response: We have added on one dataset with the MS Excel format in the revised version of manuscript.

------------------------------------------------------

------------------------------------------------------

Reviewer P:

General comments

=============

This study analyzed the 17 Dengue fever (DF) related symptoms of 177 pediatric patients to predict DF using statistical methods (mainly logistic regression).

Specific comments

=============

Major comments

---------------------

1. I believe this study is out of the interests of this journal. This study is basically a data analysis study or Dengue fever study. The study does not discuss mHealth problems. I cannot find any discussions about mHealth and mobile technologies used in the study.

Response: This study is regarding the online detection of DF on Mobile phone and related to the topic of mHealth and uHealth.

2. It is an overstatement to say “A self-assessment … may eliminate the need for a costly and time-consuming dengue laboratory test” because they used only 177 pediatric patients data.

Response: We have put one sentence in limitation about the small sample size. However, the technique using the regression method to develop an online DF detection for children is merit on the topic of mHealth and uHealth.

------------------------------------------------------
